# Population-level indicators associated with hormonal contraception use: a register-based matched case–control study

**DOI:** 10.1186/s12889-021-10512-6

**Published:** 2021-03-07

**Authors:** Elena Toffol, Oskari Heikinheimo, Anna But, Antti Latvala, Timo Partonen, Jari Haukka

**Affiliations:** 1 Department of Public Health, Clinicum, Faculty of Medicine, University of Helsinki, Helsinki, Finland; 2 Department of Obstetrics and Gynaecology, University of Helsinki and Helsinki University Hospital, Helsinki, Finland; 3 Institute of Criminology and Legal Policy, University of Helsinki, Helsinki, Finland; 4 Mental Health Unit, Department of Public Health Solutions, Finnish Institute for Health and Welfare, Helsinki, Finland; 5 Faculty of Medicine and Health Technology, Tampere University, Tampere, Finland

**Keywords:** Hormonal contraception, Finland, Municipality, Inequalities

## Abstract

**Background:**

Monitoring factors related to hormonal contraception (HC) use is essential to evaluating public health strategies and promoting access to contraception. We aimed to examine municipal social and health indicators of HC use at the population level, and to identify patterns of inequality across Finnish municipalities.

**Methods:**

We identified all women (15–49 years) with a redeemed HC prescription in Finland in 2017 (*n* = 294,445), and a control group of non-users. Municipal social and health indicators at the population level were retrieved from the nationwide Statistics and Indicator Bank. Differences between the groups across 309 municipalities were calculated, and associations of municipal-specific proportions of HC users with municipal-specific indicators were studied using LASSO (Least Absolute Shrinkage and Selection Operator) models.

**Results:**

Sociodemographic differences between HC users and non-users were non-homogenous across municipalities. Indicators positively associated with HC use included: larger population and higher proportions of population aged 16–24 years, of household-dwelling units with one person, of persons with higher education, and of divorces among those aged 25–64. Lower HC use was associated with higher proportions of Swedish-speaking population, of those aged 7–15 years, of young people not in education/training, and of household-dwelling units in overcrowded conditions. Lower HC use was also associated with indicators of outpatient and inpatient healthcare, and of municipal finances in welfare and healthcare.

**Conclusions:**

Sociodemographic differences in relation to HC use exist across Finnish municipalities. Municipal indicators of social structure, health and welfare, and investment in and use of healthcare services are related to HC use.

**Supplementary Information:**

The online version contains supplementary material available at 10.1186/s12889-021-10512-6.

## Background

Nordic countries have a traditionally long history of official national registers covering social and health information of their entire population. However, unlike other Nordic countries, where the use of hormonal contraception (HC) has long been tracked in such registers, only medications that are reimbursable from the Social Insurance Institution (SII) were recorded in Finland until 2017. Thus, monitoring the amount and pattern of use of non-reimbursable medications, including HC, was possible in Finland only on the basis of sales records. Based on these data, it was found that almost half of the Finnish women of childbearing age (15–49 years) were using HC in 2010–2013. The most commonly used methods were the pill (more than 20%) and the levonorgestrel-releasing intrauterine system (15%) [1]. However, because of the unavailability of register records, HC use in Finland could not be studied in more detail, for example in relation to individual or local sociodemographic characteristics or policy strategies.

Socioeconomic disparities are known to contribute to inequalities in health and wellbeing, including the use of healthcare services [2, 3] and lifestyle behaviours. In this context, monitoring social and health factors possibly related to HC use is essential to estimating the appropriateness of current public health strategies and policies, and to promoting equal access to contraception throughout the country. In fact, the efficacy and availability of contraception, along with adequate policies of sexual and reproductive education, as well as awareness of contraception options and programmes, have led to a considerable decline in the number of unwanted pregnancies and induced abortions, especially among adolescents and young women [4, 5]. On the other hand, not only socioeconomic and health inequalities in general are still relevant [6], but sociodemographic and health disparities, though of small size, also persist in relation to HC use in Finland [7], suggesting that strategies aimed at reducing barriers in the access to contraception could still be implemented.

In Finland there are 309 municipalities, corresponding to the local level of administration, which differ with respect to demographic, social and health indicators [8–11], including public health policies in contraception-related issues; as such, they provide an interesting setting for studying factors related to HC use on a municipal (population) level.

The recent inclusion (starting from 2017) in the Prescription Centre of retrieved HC prescriptions for the entire Finnish population, now allows for the identification of all HC users in Finland. Thus, this study aimed to explore the distributions of HC use across Finnish municipalities, to examine the social and health indicators of HC use on a municipal level, and to identify possible indicators of inequality in relation to HC use across municipalities.

## Methods

### Study population

**Fig. 1:**
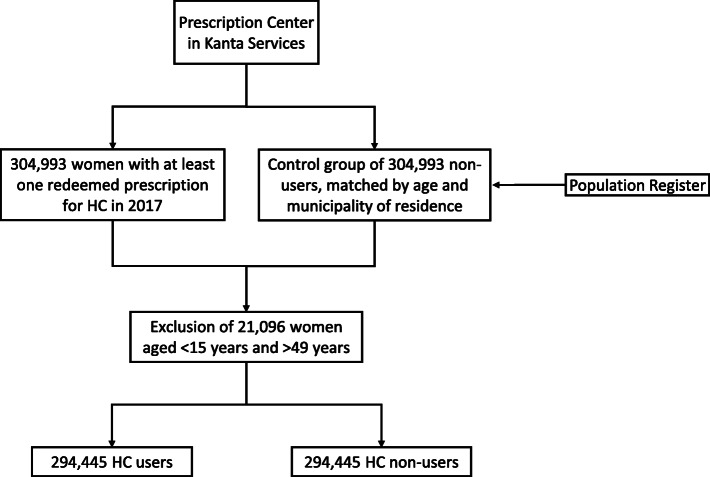
Design of the study

The original population for this study was selected on the basis of the unique personal identification number given at birth or immigration to each person living in Finland. Details of the study population and its selection are described elsewhere [7]. Briefly, all women living in Finland on 31 December 2017, with a record in the Prescription Centre of at least one redeemed HC prescription (Anatomical Therapeutic Chemical -ATC- codes: G02B, “contraceptives for topical use”; G03A, “hormonal contraceptives for systemic use”; G03HB, “antiandrogens and oestrogens”) [12] in 2017, were identified. The same-size control group consisted of women, 1:1 matched by age and municipality of residence, with no records of redeemed HC prescriptions in 2017. After exclusion of 21,096 women younger than 15 years or older than 49 years, a final group of 294,445 HC users was retained (Fig. 1).

The original study was approved by the Ethics Committee of the Faculty of Medicine, University of Helsinki (3/2018). Because this is a register-based study, no individual consent is needed.

### The registers

The Prescription Centre is a centralised database in the Kanta Services [13], which includes information on prescribed and redeemed medications for each person living in Finland (e.g., the product ATC code, date of prescription and purchase, prescribed amount in defined daily dose).

Information on sociodemographic characteristics of all the study members on 31 December 2017 (age, municipality of residence, civil status, socioeconomic group, highest level of education, annual income) was obtained through Statistics Finland, from the Population Register Centre, which includes basic information of all Finnish citizens and foreign citizens residing permanently in Finland.

### Municipal-level data

Indicators on population, health, welfare, functioning, services and resources at the municipal level (expressed as absolute numbers, means, rates or indexes for each municipality) were retrieved from the Sotkanet Statistics and Indicator Bank, an information service of the Finnish Institute for Health and Welfare (THL) that includes welfare and health data from 1990 onwards on all Finnish municipalities [14]. Only indicators available for the female population of each municipality as of 2017 were selected, resulting in the 119 indicators listed in an additional table (Additional Table 1 in Additional file 1).

### Statistical analyses

The proportions of HC users in all 309 municipalities were calculated. Differences in sociodemographic characteristics between HC users and non-users in each municipality of residence were examined via chi-squared test or t-test, as appropriate. Based on previous findings of sociodemographic factors related to HC use [7, 15, 16], the following sociodemographic variables were examined: socioeconomic group (− defined as “a person’s position in society, ( …) based on data on the person’s main type of activity, occupation, occupational status and industry” [17] – self-employed, upper-level employees, lower-level employees, manual workers, students, pensioners, others, unknown, missing); education (upper secondary/post-secondary non-tertiary, short-cycle tertiary, bachelor, master/doctoral, missing); and income (as a continuous variable). For these analyses two-tailed *p*-values < 0.05 were considered statistically significant.

We further conducted analyses on municipal-specific rates of HC use and Sotkanet indicators, with the aim of identifying possible predictors of HC use on a municipal level. To this end, the associations between the proportion of HC users in aggregated municipality data (treated as the dependent variable) and several sociodemographic and welfare indicators (as independent variables) were studied using the LASSO (Least Absolute Shrinkage and Selection Operator) [18] logistic regression model with an α-parameter of 0.9, which made the model an elastic net. The model’s λ was determined using cross-validation; one standard error over minimum λ was used in the final model. We applied LASSO to indicator values dichotomised by weighted median values. Of the 119 indicators included as independent variables in the initial model (Additional Table 1 in Additional file 1), the 13 dichotomised indicators reported in Table 2 were selected for the final model. Sensitivity analyses were conducted by using continuous values of municipal indicators in the LASSO model (Additional Table 2 in Additional file 2). An elastic net was used to carry out both, variable selection and regularization, in order to enhance the prediction accuracy and interpretability of the resulting model [19].

All the analyses were performed with R version 3.5.1 [20].

## Results

A total of 294,445 women (25.8% of Finnish female population aged 15–49 years) were HC users in Finland in 2017. Their basic characteristics are described in Table 1.

**Table 1:** Basic characteristics of women using HC and their matched controls

	HC users (*n* = 294,445)	HC non-users (*n* = 294,445)
	mean (SD) / n (%) / median (IQR range)	
**Age,** years	28.9 (8.6)	28.9 (8.6)
**Civil status**		
Unmarried	211,519 (71.8)	192,488 (65.4)
Married	65,710 (22.3)	85,535 (29.0)
Divorced	16,518 (5.6)	15,241 (5.2)
Widowed/Other	698 (0.3)	1181 (0.4)
**Highest education level**		
Upper secondary/Post-secondary non-tertiary	142,373 (48.4)	134,095 (45.6)
Short-cycle tertiary	8187 (2.8)	7229 (2.5)
Bachelor’s degree	60,507 (20.5)	51,710 (17.6)
Master’s, doctoral or equivalent	34,004 (11.6)	31,390 (10.6)
Missing (including e.g., missing information on education other than of primary school level, school dropouts)	49,374 (16.8)	69,979 (23.8)
**Socio-economic group**		
Self-employed	10,070 (3.4)	10,708 (3.6)
Upper-level employees	37,177 (12.6)	34,823 (11.8)
Lower-level employees	104,890 (35.6)	86,057 (29.2)
Manual workers	45,119 (15.3)	42,264 (14.4)
Students	56,607 (19.2)	63,946 (21.7)
Pensioners	3748 (1.3)	6730 (2.3)
Others	22,358 (7.6)	28,774 (9.8)
Unknown	13,626 (4.6)	16,964 (5.8)
Missing	850 (0.3)	4179 (1.4)
**Income**, €	19,580 (14756)	17,042 (16254)

*HC* Hormonal Contraception

**Fig. 2:**
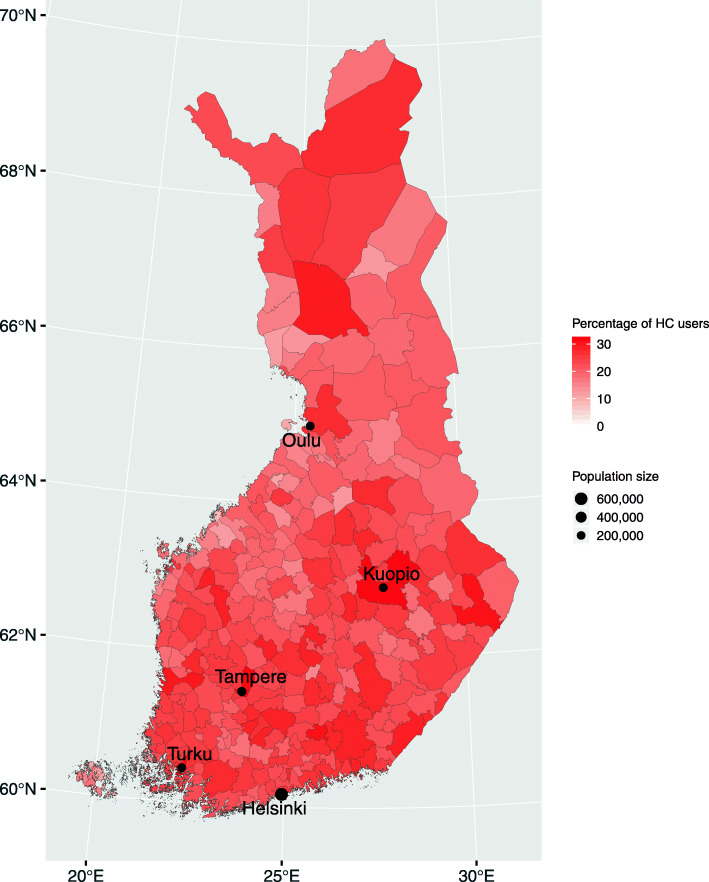
Proportions of HC use across 309 Finnish municipalities. (map created with R software version 3.5.1, URL http://www.rstudio.com/)

The proportions of HC users across the 309 municipalities are illustrated in Fig. 2. The rates of HC use ranged between 0 and 15% in municipalities located mostly in Swedish-speaking regions (e.g., the Åland Islands and Ostrobothnia, western coast), and over 30% in areas of the larger cities such as the Kuopio, Turku and Tampere regions. The proportion of HC use in the capital area ranged between 22.9% (Vantaa) and 26.2% (Helsinki).

**Fig. 3:**
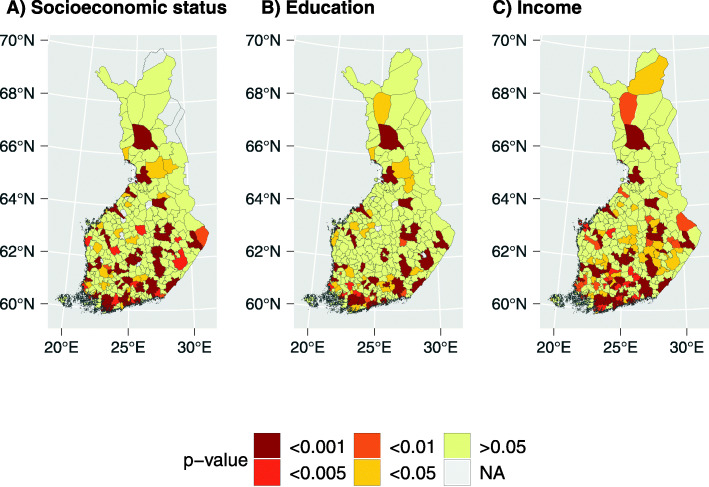
Significance levels of sociodemographic differences between HC users and non-users across 309 Finnish municipalities. **a** Socioeconomic status; **b** Education; **c** Annual income. *P*-values are from chi-squared test and t-test. (maps created with R software version 3.5.1, URL http://www.rstudio.com/)

Figure 3 shows the significance levels for differences between HC users and non-users across municipalities (A: socioeconomic status; B: education; C: income). Sociodemographic differences were non-homogenous across districts: they were not significant in most of the municipalities, with the exception of the capital area (Helsinki, Espoo and Vantaa) and the areas of the larger cities. Specifically, differences in education and socioeconomic levels were more significant in the Northern regions of Rovaniemi, Oulu, Kajaani, Kokkola, in the Eastern regions of Kuopio, Joensuu, Savonlinna and Mikkeli, and more generally in the Southern regions of Finland. In addition, socioeconomic differences were detectable at a significant level in a large number of regions concentrated in Central and Western Finland.

**Fig. 4:**
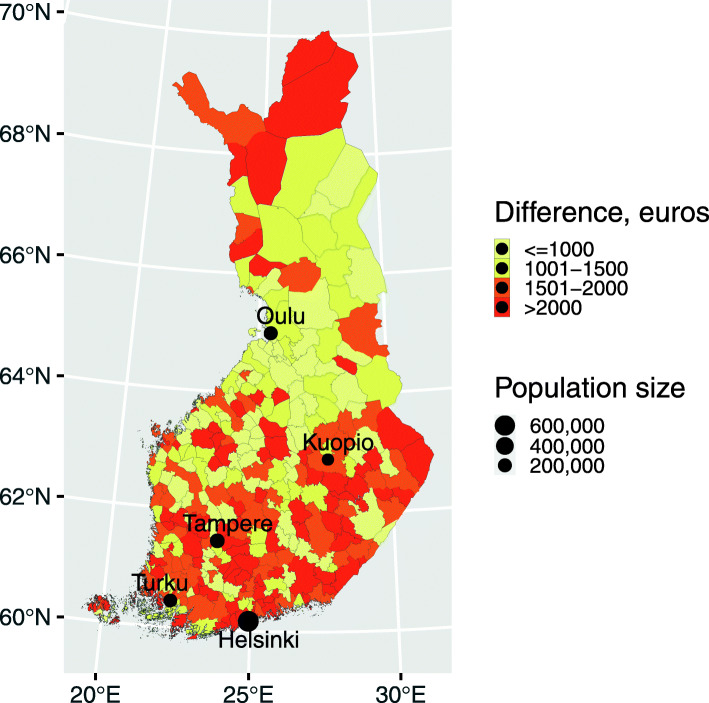
Annual income difference (in euros) between HC users and non-users across 309 Finnish municipalities. (map created with R software version 3.5.1, URL http://www.rstudio.com/)

The pattern of distribution of annual income inequalities was similar to that of socioeconomic differences, but encompassing a larger number of regions in Central and Southern Finland. The largest differences in annual income between HC users and non-users (over 2000 €) were found in Lapland and in the capital area, while medium-level differences (1500–2000 €) were detectable mostly in Central Finland (Fig. 4).

In analyses of aggregated data with the LASSO model, the proportion of HC users was higher in municipalities with larger populations (as indicated by the population at year end, and by the numbers of household-dwelling units, families and live births) and with more persons with higher education qualifications, but lower in municipalities with more household-dwelling units living in overcrowded conditions (defined as “a dwelling with more than one person per room when the kitchen is not included in the number of rooms”) [14] (Table 2).

**Table 2:** Indicators associated with municipality level hormonal contraception (HC) use in Finland

Indicator	Median	Weighted median	Range	OR
**Population, Social environment of population**				
Population at year end	3095	36,700	144 to 334,171	1.00950
Total number of household-dwelling-units	2810	37,355.4	157 to 325,319	1.00001
Proportion of household-dwelling units living in overcrowded conditions (% of all household-dwelling-units)	8.4	8.2	5.0 to 19.3	0.91961
Proportion of household-dwelling-units with one person (% of all household-dwelling-units)	40.5	43.6	19.9 to 51.4	1.07046
Families, total	1695	19,325.9	83 to 156,620	1.00062
Persons with higher education qualifications, aged 15 and over, as % of total population of same age	25.8	36.0	12.6 to 58.6	1.02204
Number of live births	26	352.9	1 to 3320	1.06115
**Cost-effectiveness indicators in social welfare and health care, Social assistance, Services and resources**				
Basic social assistance during year, euro per capita (real prices)	60	114.2	0 to 231	1.00301
Prenatal clinic visits in primary health care per 1000 women aged 15–44	931	789.2	20 to 2605	0.98635
Psychiatric inpatient care, periods of care per 1000 inhabitants	5.9	6.4	1.0 to 31.5	1.04426
**Municipal finances in social and health care**				
Operating net expenditure on oral health care, euro per capita	88.5	74.3	0.0 to 177.0	0.99784
Operating net expenditure on services in support of employment, euro per capita	44.5	99.7	−3.5 to 223.2	1.01693
Operating net expenditure on other services for the disabled, euro per capita	171	158.3	0 to 670	0.99425

Odds ratios (*OR*) are based on logistic regression model with proportion of women using HC as outcome, weighted by population size. Indicators dichotomized by weighted median, above median compared to below median. Median and range refer to the median, minimum and maximum value of the corresponding indicator across 309 Finnish municipalities

Sensitivity analyses using continuous variables additionally indicated that municipalities with higher proportions of population aged 16–24 years, of household-dwelling units with one person, and of divorces among those aged 25–64 years had higher proportions of HC users. On the other hand, higher proportions of population aged 7–15 years, of Swedish-speaking populations, and of those aged 17–24 years not in education or training were negatively associated with HC use (Additional Table 2 in Additional file 2).

HC use was less likely associated with indicators of outpatient and inpatient healthcare, and of cost-effectiveness and municipal finances in social welfare and healthcare. The main exceptions was the higher likelihood of HC use in relation to higher number of psychiatric inpatient care periods and to higher municipal expenses supporting employment and basic social assistance (Table 2 and Additional Table 2 in Additional file 2).

A total of four variables (“Household-dwelling units living in overcrowded conditions”, “Household-dwelling units with one person”, “Prenatal clinic visits in primary healthcare per 1000 women aged 15–44” and “Operating net expenditure on oral healthcare”) were included in both final LASSO models (Table 2 and Additional Table 2 in Additional file 2).

Indicators of disposable income, economic dependency ratio, population structure, availability and use of specialised care, entitlement to disability benefits, rates of crime suspects, distribution of employment and occupations in the municipality, investment in social and employment services, mortality, morbidity and disability were not related to the proportion of HC users (Additional Table 1 in Additional file 1).

## Discussion

More than a quarter of childbearing-age women used HC in Finland in 2017. Sociodemographic differences in HC use were not homogeneous across municipalities. In addition, the use of contraception was related to a small number of general indicators of the population, health and welfare, as well as to municipal finances in and use of healthcare services and resources.

The 26% rate of HC use in Finland only appears low relative to previously reported figures based on sales data from Nordic countries. In fact, Lindh et al. reported a 30–40% HC use in each of the Nordic countries (Denmark, Iceland, Norway, Finland and Sweden) in 2013 [1]. However, while records of HC use are available in Finnish registers from 2017 onwards, long-acting reversible contraceptive (LARC) methods can be used for several years after insertion. Thus, it is likely that our data, while almost completely capturing oral contraceptive users, in fact underestimates the real proportion of women using LARC methods in Finland, which is likely to be approximately 20%. On this regard, it is worth noticing that a growing number of municipalities in Finland are developing a free-of-charge contraception program for young women, who cannot be completely identified through records of redeemed prescriptions, and thus were not included in our group of HC users.

Socioeconomic and sociodemographic inequalities remain a recognized determinant of wellbeing, morbidity and mortality in many countries, including Finland [3, 21]. Public health policies and strategies targeting the more disadvantaged groups and aiming to facilitate their access to healthcare services and promote healthy lifestyle and behaviour, including reproductive choices and health, may result in a reduction of such inequalities. Sociodemographic differences are widely reported also between HC users and non-users [15, 16, 22–25]. Similarly, in Finland we previously reported that the proportion of HC use is higher among single and divorced women, and in those with higher education, socioeconomic and income levels. Additionally, we found about 60% of all HC users in Finland to be younger than 30 years [7]. In line with those previous findings of ours, in the current study the proportions of HC users appeared lower in municipalities with higher proportions of those aged 17–24 years not in education or training, but higher in municipalities with higher proportions of the population aged 16–24 years and of persons with higher education, with more household-dwelling units with one person (possibly reflecting unmarried and divorced women), and divorces among those aged 25–64 years. The additional finding of more HC users in relation to municipal investment in services supporting employment may further explain previous findings of higher HC use among employed women. In the current societal and demographic context, our results potentially indicate a trend for increasing contraception use in Finland as a reflection of ongoing trends in growing urbanisation, drops in birth rates, as well as increasing levels of female education. However, it must be mentioned that although the LASSO models do not provide any information on the size of the observed associations, the obtained Odds Ratios (OR) were rather small.

The socioeconomic and demographic differences between HC users and non-users were more obvious around the larger cities in North and Central Finland, as well as in the capital area. Accordingly, municipalities with larger populations were also those with more HC users. This figure is likely to reflect the age structure of the population across municipalities in Finland, where the larger cities are those with higher proportions of population comprised by young women. On the contrary, smaller municipalities have higher proportions of population comprised of individuals aged 65 or older [14]. The lowest rates of HC use were found in municipalities belonging to the Swedish-speaking areas (e.g., Åland islands and Ostrobothnia); we consistently found lower ORs of HC use in municipalities with more Swedish-speaking populations. It is of note that while these figures possibly reflect different patterns of contraception use (e.g., wider use of copper intrauterine devices [1], which is not captured by our study), they partially inversely correspond to induced abortion rates in Finland, which are the highest in the Åland islands [26].

Despite the non-homogeneous distribution of socioeconomic differences in relation to HC use across municipalities, in general, the overall rate of contraception use in Finland was fairly high and comparable to that of other developed countries [1, 27, 28]. Furthermore, municipal indicators of disposable income, at-risk-of-poverty rate, and economic dependency ratio were not associated with the proportions of HC use, suggesting that current public health policies guarantee adequate access to contraception to women of different socioeconomic and demographic conditions across the country. Additionally, we did not find any associations with a large number of indicators of social structure, such as nationality (as indicated by the native language proxy), employment (with the exception of municipal expenditure on services supporting employment) and unemployment levels, and occupation type. These outcomes further suggest a general good access to contraception, irrespective of cultural or social structure. Similarly, our preliminary finding of a lack of association with the number of crime suspects in those younger than 20 years suggests good contraception use among individuals with behavioural and criminal problems, who are generally at risk of contraception non-use and unintended pregnancy [29]. However, contraception use in relation to criminal and other possibly impulsive behaviour needs to be studied in more detail.

Contrary to our previous findings of lower HC use in women with a recent care episode for psychiatric disorders [7], most of the municipal indicators of psychiatric as well as somatic care, along with indicators of entitlement to health benefits, were not associated with the proportion of HC use. Rather, when using dichotomised indicators only, a higher number of psychiatric inpatient care periods was associated with a higher proportion of HC use.

It warrants attention that the proportions of HC users were lower in those communities with higher investment/expense on primary healthcare. In particular, municipalities with higher numbers of prenatal and family-planning clinic visits in primary healthcare had lower HC use. These results are, however, only potentially contradictory. It is in fact plausible that municipalities with higher investment in primary healthcare have also already implemented a better-developed policy on contraception (including LARC methods) free-of-charge [30]. Because contraception free-of-charge is not recorded in the Prescription Centre, we were not able to capture these HC users. On the one hand, the lack of a national policy on this issue contributes to maintaining disparities across communities. However, the increasing number of communities offering contraception free-of-charge is potentially limiting the possible negative impact of socioeconomic factors on HC use. This is of particular importance in relation to the more expensive methods, such as LARC methods, where costs may still represent a barrier to access. This is confirmed by the observation that inclusion of LARC methods in the contraception free-of-charge programme has resulted in a local increase in the use of such methods and decline of municipal abortion rates [30]. Similarly, the negative association between HC use and a few municipal healthcare indicators, such as the number of clients of outpatient medical care in primary healthcare or the duration of periods of care with surgical procedures, may reflect both a lower use of contraception in women with more severe health problems as well as the increasing prescription of LARC methods in connection with medical or surgical procedures.

Even though the two LASSO models identified different indicators when using dichotomised rather than continuous variables, the selected indicators were consistent markers of socioeconomic and demographic structure, as well as of municipal investment in health and welfare. The LASSO is generally used for prediction, and its interpretation may be challenging. Therefore, our LASSO model should be interpreted more as a descriptive tool showing associations. Significance testing with the model is not feasible because the data are utilised several times during model selection.

### Strengths and limitations

This study has a number of limitations. First, the original population was selected on the basis of register records of prescriptions, which as such do not guarantee the actual contraceptive use. However, because HC is not reimbursed by the SII, it is likely that the majority of women who purchased the drug did in fact use it. On the other hand, it is likely that a number of HC non-users were in fact using HC, for example because of a prescription redeemed, or hormonal intrauterine devices/implants inserted, during the previous years. This especially concerns LARC methods, which can be used for several years after insertion. However, we were able to include among HC users women who had a prescription made before 2017, but redeemed in 2017. Even if pharmacy data and prescription registers in Nordic countries are accurate and complete [31], because of the only recent inclusion of HC records in the Prescription Centre, completeness of the data cannot be guaranteed. Moreover, we were not able to identify women who had received contraception free-of-charge from the community/family planning services. Additionally, this study does not cover the use of non-hormonal contraception, such as condoms or copper intrauterine devices, which remain widely used in Finland. In principle, the LASSO method does not allow calculation of confidence intervals of estimates because the data are used several times in the cross-validation process. Therefore, the reported ORs merely describe point estimates of the direction of the associations.

Despite the above limitations, strengths of the study include the representativeness of the population, and the use of register data, which have proven good validity and reliability [32]. Additionally, use of official indicators on population health and welfare guarantee extensive valid and reliable information of the municipality characteristics.

## Conclusions

More than a quarter of childbearing-age women use HC in Finland. Sociodemographic inequalities are observable across Finnish municipalities. A number of municipal indicators of health and welfare, as well as of municipal investment in and use of healthcare services and resources are related to the proportion of HC users.

## Supplementary Information

**Additional file 1: Additional Table 1.** Municipal indicators used in the preliminary LASSO models.

**Additional file 2: Additional Table 2.** Indicators associated with municipal-level hormonal contraception (HC) use in Finland.

## Data Availability

The data that support the findings of this study are available from Statistics Finland, the Social Insurance Institution and the Finnish Cancer Registry, but restrictions apply to the availability of these data, which were used under license for the current study, and so are not publicly available. Data are, however, available from the authors upon reasonable request and with permission of FinData (https://www.findata.fi/en/).

## Abbreviations

ATC: Anatomical Therapeutic Chemical
HC: Hormonal contraception
LARC: Long-acting reversible contraceptive
LASSO: Least Absolute Shrinkage and Selection Operator
OR: Odds Ratio
SII: Social Insurance Institution
THL: Finnish Institute for Health and Welfare
