# Supplementary material for: Population-level indicators associated with hormonal contraception use: a register-based matched case–control study
Source: BMC Public Health. 2021 Mar 7;21:465. doi: 10.1186/s12889-021-10512-6 (PMC7938490; doi:10.1186/s12889-021-10512-6)
Supplement: Supplementary file 1 — Additional file 1: Additional Table 1. Municipal indicators used in the preliminary LASSO models. [file 12889_2021_10512_MOESM1_ESM.docx]

**Additional Table 1. Municipal indicators used in the preliminary LASSO model.**

| **Indicator** | **Median** | **Range** | **Median absolute deviation** |
| --- | --- | --- | --- |
| **Population, Social environment of population, General indicators of health and welfare** |  |  |  |
| *Number of live births* | *26* | *1 to 3 320* | *28.2* |
| Population density, population/km2 | 10.6 | 0.2 to 2 965.2 | 10.1 |
| Number of deaths | 38 | 0 to 2 758 | 35.6 |
| *Population at year end* | *3,095* | *144 to 334 171* | *2 889.6* |
| Population aged 20-24, year-end total | 97 | 5 to 25 667 | 96.4 |
| Population aged 25-29, year-end total | 113 | 3 to 32 917 | 118.6 |
| Population aged 30-34, year-end total | 139 | 4 to 29 118 | 143.8 |
| Population aged 35-39, year-end total | 151 | 3 to 24 449 | 155.7 |
| Population aged 40-44, year-end total | 158 | 4 to 19 703 | 161.6 |
| Population aged 45-49, year-end total | 177 | 6 to 19 513 | 172.0 |
| Population aged 0-6 as % of the total population | 6.6 | 2.1 to 15.3 | 1.8 |
| Population aged 0-15 as % of total population | 16.0 | 8.0 to 36.1 | 3.6 |
| *Population aged 7-15 as % of total population* | *9.5* | *4.3 to 20.9* | *2.1* |
| *Population aged 16-24 as % of total population* | *7.5* | *3.3 to 14.6* | *1.5* |
| Population aged 25-64 as % of total population | 46.9 | 38.6 to 55.8 | 3.1 |
| Population aged 18 and over as % of total population | 81.7 | 60.7 to 90.8 | 3.7 |
| *Families, total* | *1,695* | *83 to 156 620* | *1 587.9* |
| Single parent families, as % of all families with children | 18.8 | 4.6 to 28.3 | 4.0 |
| Families with children, as % of all families | 34.8 | 20.3 to 62.6 | 6.5 |
| *Household-dwelling-units, total* | *2,810* | *157 to 325 319* | *2 542.7* |
| *Household-dwelling-units with one person, as % of all household-dwelling-units* | *40.5* | *19.9 to 51.4* | *5.9* |
| Household-dwelling-units with at least one person aged under 18, as % of all household-dwelling-units | 20.1 | 10.6 to 48.1 | 5.0 |
| *Household-dwelling-units living in overcrowded conditions, as % of all household-dwelling-units* | *8.4* | *5.0 to 19.3* | *1.6* |
| Household-dwelling-units with children living in overcrowded conditions, as % of all households with children | 30.6 | 17.6 to 62.8 | 5.5 |
| Native language other than Finnish, Swedish or Sami per 1000 inhabitants, number | 23.1 | 3.0 to 159.2 | 13.8 |
| Swedish-speaking population at year end | 8 | 0 to 18,741 | 10.4 |
| *Swedish-speaking population as % of total population at year end* | *0.2* | *0.0 to 92.2* | *0.3* |
| *Divorces among those aged 25-64 per 1000 married persons of same age* | *14.7* | *0.0 to 44.8* | *6.1* |
| Persons with upper secondary education, as % of total population aged 20 and over | 41.4 | 23.5 to 48.5 | 2.8 |
| *Persons with higher education qualifications, as % of total population aged 20 and over* | *25.8* | *12.6 to 58.6* | *5.9* |
| *Those aged 17-24 not in education or training, as % of total population of same age* | *6.8* | *0.0 to 41.7* | *2.7* |
| Students, as % of total population | 6 | 2 to 11 | 1.3 |
| Employed, as % of total population | 36.8 | 27.6 to 54.4 | 5.0 |
| Employed, aged 18-24, number | 76 | 5 to 19 066 | 77.1 |
| Wholesale and retail trade; Transportation and storage; Accommodation and food service activities, % of total active labour force | 18.0 | 6.9 to 33.6 | 3.3 |
| Information and communication, % of total active labour force | 1.0 | 0.0 to 5.7 | 0.6 |
| Financial and insurance activities, % of total active labour force | 1.8 | 0.0 to 5.4 | 0.7 |
| Real estate activities, % of total active labour force | 0.7 | 0.0 to 2.9 | 0.5 |
| Agriculture, forestry and fishing, % of total active labour force | 4.7 | 0.1 to 20.9 | 4.3 |
| Professional, scientific and technical activities; Administrative and support service activities, % of total active labour force | 8.9 | 0.0 to 18.1 | 2.7 |
| Public administration and defence, compulsory social security; Education; Human health and social work activities, % of total active labour force | 47.1 | 32.7 to 58.8 | 5.3 |
| Manufacturing, % of total active labour force | 6.6 | 0.0 to 30.0 | 3.1 |
| Other service activities, % of total active labour force | 6.4 | 2.3 to 14.5 | 1.2 |
| Construction, % of total active labour force | 1.1 | 0.0 to 3.3 | 0.6 |
| Mining and quarrying; Electricity, gas, steam and air conditioning supply; Water supply, sewerage, waste management and remediation activities, % of total active labour force | 0.4 | 0.0 to 3.7 | 0.3 |
| Crime suspects aged 15-17 per 1000 inhabitants of same age | 10.4 | 0.0 to 120.5 | 15.4 |
| Crime suspects aged 18-20 per 1000 inhabitants of same age | 20.4 | 0.0 to 440.3 | 30.3 |
| Economic dependency ratio | 165.5 | 86.8 to 254.5 | 36.2 |
| General at-risk-of-poverty rate for the municipality | 13.7 | 2.9 to 23.4 | 3.9 |
| Gini coefficient, disposable income | 23.8 | 20.4 to 49.2 | 1.5 |
| Refugees received per 100,000 inhabitants | 0 | 0 to 1,726 | 0 |
| **Population health status** |  |  |  |
| Average overall pensions, pensions in one’s own right, euro per month | 1,291 | 1 109 to 2 082 | 108.2 |
| Disability pension recipients, as % of aged 16-64 | 7.9 | 2.1 to 17.9 | 2.7 |
| Mortality per 100 000 inhabitants | 1,233 | 0 to 4 110 | 481.4 |
| **Cost-effectiveness indicators in social welfare and health care, Social assistance, Services and resources** |  |  |  |
| *Basic social assistance during year, euro per capita (real prices)* | *60* | *0 to 231* | *31.1* |
| SII’s index for entitlement to special reimbursements, age standardised | 103.6 | 71.1 to 129.1 | 12.3 |
| SII’s disability index, age standardized | 114.1 | 32.9 to 241.9 | 30.3 |
| SII’s morbidity index, age standardized | 107.7 | 55.5 to 162.1 | 16.0 |
| THL’s morbidity index, age-standardized | 105.3 | 49.9 to 174.7 | 18.8 |
| THL’s morbidity index, not age-standardized | 115.6 | 52.9 to 215.3 | 26.2 |
| SII’s mortality index, age standardized | 102.2 | 55.9 to 145.5 | 11.7 |
| Outpatients and inpatients in primary health care, % of population | 90.5 | 52.5 to 101.3 | 5.8 |
| *Clients of outpatient medical care in primary health care per 1000 residents* | *651* | *263 to 877* | *98.0* |
| Primary health care outpatient clients per 1000 inhabitants | 904 | 506 to 1 021 | 59.5 |
| Outpatient physician visits in primary health care (all methods of contact) per 1000 inhabitants | 2,695 | 525 to 6 462 | 859.9 |
| Outpatient visits in specialised somatic health care per 1000 inhabitants | 1,566 | 839 to 4 709 | 338.8 |
| Outpatient specialised health care visits per 1000 inhabitants | 168.5 | 55.2 to 698.8 | 50.1 |
| Outpatient visits in specialised health care, obstetrics and gynaecology, per 1000 women | 190.4 | 37.6 to 803.3 | 54.0 |
| Somatic outpatient visits in specialised health care in total for those aged 18 and over per 1000 persons of same age | 1,722 | 888 to 5 350 | 343.8 |
| Family-planning visits in primary health care per 1000 inhabitants | 36 | 0 to 116 | 22.2 |
| Family-planning-clinic visits (physicians) in primary health care per 1000 inhabitants | 11 | 0 to 63 | 10.4 |
| *Family-planning-clinic visits (practitioners other than physicians) in primary health care per 1000 inhabitants* | *25* | *0 to 93* | *16.3* |
| *Prenatal clinic visits in primary health care per 1000 women aged 15-44* | *931* | *20 to 2 605* | *235.7* |
| Proportion of women invited to breast cancer screening (mammography) who attended, % | 84.7 | 68.9 to 97.4 | 3.0 |
| Hospital care, periods of care per 1000 inhabitants | 281 | 140 to 515 | 75.2 |
| Hospital care, average length of stay | 5.9 | 2.7 to 30.2 | 1.8 |
| Hospital care, care days per 1000 inhabitants | 1,577 | 455 to 7 920 | 704.8 |
| Hospital care, patients per 1000 inhabitants | 159.4 | 97.7 to 225.9 | 24.6 |
| Specialised somatic inpatient health care, periods of care per 1000 inhabitants | 152.3 | 97.9 to 247.3 | 29.8 |
| Specialised somatic inpatient health care, average length of stay | 3.4 | 2.3 to 8.4 | 0.5 |
| Specialised somatic inpatient health care, care days per 1000 inhabitants | 524 | 321 to 1 972 | 132.0 |
| Specialised somatic inpatient health care, patients per 1000 inhabitants | 103 | 70 to 152 | 16.0 |
| Specialised somatic inpatient health care, periods of care for those aged 18 and over per 1000 persons of same age | 174 | 116 to 292 | 32.6 |
| Specialised somatic inpatient health care, care days for those aged 18 and over per 1000 persons of same age | 588 | 382 to 2,352 | 141.6 |
| Specialised somatic inpatient health care, patients aged 18 and over per 1000 persons of same age | 117.9 | 84.1 to 167.7 | 18.2 |
| *Psychiatric inpatient care, periods of care per 1000 inhabitants* | *5.9* | *1.0 to 31.5* | *2.8* |
| Psychiatric inpatient care, average length of stay | 22.5 | 1.0 to 401.8 | 11.1 |
| Psychiatric inpatient care, care days per 1000 inhabitants | 137.3 | 2.1 to 768.2 | 83.0 |
| Periods of care with surgical procedures per 1000 inhabitants | 55.4 | 23.9 to 86.4 | 10.8 |
| *Periods of care with surgical procedures, average length* | *5.4* | *2.7 to 16.5* | *1.2* |
| *Periods of care with surgical procedures, care days per 1000 inhabitants* | *293* | *104 to 1 208* | *74.1* |
| Periods of care with surgical procedures, patients per 1000 inhabitants | 50.9 | 23.0 to 77.2 | 9.8 |
| Age- and sex-standardised index for periods of care for patients in hospital care | 109 | 57 to 166 | 18.5 |
| Age- and sex-standardised index for care days for patients in hospital care | 94.8 | 31.0 to 255.8 | 28.5 |
| Age- and sex-standardised index for periods of care for patients in specialised somatic inpatient health care | 100.1 | 67.6 to 159.9 | 15.4 |
| Age- and sex-standardised index for care days for patients in specialised somatic inpatient health care | 86.1 | 53.1 to 288.1 | 16.6 |
| Age- and sex-standardised index for periods of care for patients in psychiatric inpatient care | 90.3 | 8.2 to 340.4 | 35.9 |
| Age- and sex-standardised index for care days for patients in psychiatric inpatient care | 77.6 | 2.3 to 351.1 | 45.7 |
| Entitled to special refunds on medicines as % of total population | 30.0 | 15.2 to 46.8 | 6.4 |
| Entitled to special refunds on medicines in those aged 25-64, as % of total population of the same age | 24.2 | 13.9 to 39.4 | 4.6 |
| Entitled to special refunds on medicines for diabetes, as % of total population | 6.5 | 1.8 to 12.5 | 2.1 |
| Entitled to special refunds on medicines for hypertension in those aged 25 and over, as % of total population of same age | 14.2 | 5.5 to 27.2 | 4.9 |
| **Municipal finances, Municipal finances in social and health care** |  |  |  |
| Tax revenue, euro per capita | 3,436 | 2,196 to 6 701 | 511.5 |
| Total operating net municipal expenditure, euro per capita | 6,029 | 4,178 to 9 236 | 761.9 |
| Operating net expenditure on primary health care (including oral health care), euro per capita | 715 | 0 to 2 198 | 235.0 |
| Operating net expenditure on ward care in primary health care, euro per capita | 192 | 0 to 942 | 116.7 |
| *Operating net expenditure on oral health care, euro per capita* | *88.5* | *0.0 to 177.0* | *20.8* |
| *Operating net expenditure on outpatient care in primary health care (excl. oral health care), euro per capita* | *420* | *0 to 1 220* | *138.5* |
| Operating net expenditure on specialised health care, euro per capita | 1,280 | 0 to 1 948 | 186.2 |
| Operating net expenditure on the municipal social services and health care (excl. early childhood education and care), euro per capita | 3,599 | 1 056 to 5 808 | 746.3 |
| Operating net expenditure on special services for substance abusers, euro per capita | 14.1 | 0.0 to 164.9 | 13.1 |
| Operating net expenditure on municipal other social services and health care activities, euro per capita | 128 | -106 to 342 | 68.9 |
| Operating net expenditure on environmental health care, euro per capita | 23.7 | -17.9 to 78.7 | 14.7 |
| *Operating net expenditure on services in support of employment, euro per capita* | *44.5* | *-3.5 to 223.2* | *48.9* |
| Operating net expenditure on home care services, euro per capita | 220 | 0 to 1 555 | 102.2 |
| *Operating net expenditure on other services for the disabled, euro per capita* | *171* | *0 to 670* | *69.2* |
| Operating net expenditure on other non-institutional services for children and families, euro per capita | 36.0 | -19.6 to 316.4 | 24.6 |
| Operating net expenditure on non-institutional services in child welfare, 1000 euro | 41.4 | -17.1 to 170.0 | 28.5 |
| Operating net expenditure on institutional and family care in child welfare, euro per capita | 90.5 | -8.6 to 280.2 | 61.2 |

SII, Social Insurance Institution of Finland; THL, Finnish Institute for Health and Welfare.

Significant indicators are in italic.
