# Supplementary material for: Population-level indicators associated with hormonal contraception use: a register-based matched case–control study
Source: BMC Public Health. 2021 Mar 7;21:465. doi: 10.1186/s12889-021-10512-6 (PMC7938490; doi:10.1186/s12889-021-10512-6)
Supplement: Supplementary file 2 — Additional file 2: Additional Table 2. Indicators associated with municipal-level hormonal contraception (HC) use in Finland. [file 12889_2021_10512_MOESM2_ESM.docx]

**Additional Table 2. Continuous Indicators associated with municipal-level hormonal contraception (HC) use in Finland.**

| **Indicator** | **Median** | **Weighted median** | **Range** | **OR** |
| --- | --- | --- | --- | --- |
| **Population, Social environment of population** |  |  |  |  |
| Proportion of the population aged 7-15 years | 9.5 | 9.20 | 4.3 to 20.9 | 0.9998757 |
| Proportion of the population aged 16-24 years | 7.5 | 10.47 | 3.3 to 14.6 | 1.0159705 |
| Proportion of the population aged 17 – 24 years not in education or training | 6.8 | 7.10 | 0.0 to 41.7 | 0.9992679 |
| Proportion of household-dwelling units living in overcrowded conditions (% of all household-dwelling-units) | 8.4 | 8.20 | 5.0 to 19.3 | 0.9725128 |
| Proportion of household dwelling-units with one person (% of all household-dwelling-units) | 40.5 | 43.60 | 19.9 to 51.4 | 1.0061058 |
| Proportion of Swedish-speaking population | 0.2 | 0.50 | 0.0 to 92.2 | 0.9970814 |
| Number of divorces among those aged 25-64 (per 1000 married persons of same age) | 14.7 | 19.00 | 0.0 to 44.8 | 1.0067454 |
| **Cost-effectiveness indicators in social welfare and health care, Social assistance, Services and resources** |  |  |  |  |
| Number of family-planning-clinic visits in primary health care (per 1000 inhabitants) | 25 | 21.00 | 0 to 93 | 0.9993155 |
| Number of prenatal clinic visits in primary health care (per 1000 women aged 15 – 44) | 931 | 789.19 | 20 to 2 605 | 0.9999771 |
| Periods of care with surgical procedures (average length, days) | 5.4 | 5.60 | 2.7 to 16.5 | 0.9917174 |
| Periods of care with surgical procedures (number of days/1000 inhabitants) | 293 | 297.54 | 104 to 1 208 | 0.9997549 |
| Number of clients of outpatient medical care in primary health care | 651 | 520.40 | 263 to 877 | 0.9997273 |
| **Municipal finances in social and health care** |  |  |  |  |
| Operating net expenditure on oral health care, euro per capita | 88.5 | 74.32 | 0.0 to 177.0 | 0.9995496 |
| Operating net expenditure on outpatient care in primary health care, euro per capita | 420 | 318.26 | 0 to 1 220 | 0.9999429 |

Odds ratios (OR) based on logistic regression model with proportion of women using HC as outcome, weighted by population size. Indicators used as continuous variables. Median and range refer to the median, minimum and maximum value of the corresponding indicator across 309 Finnish municipalities.
